# Temperature-sensitive PSII: a novel approach for sustained photosynthetic hydrogen production

**DOI:** 10.1007/s11120-016-0232-3

**Published:** 2016-03-07

**Authors:** Vinzenz Bayro-Kaiser, Nathan Nelson

**Affiliations:** Department of Biochemistry, The George S. Wise Faculty of Life Sciences, Tel Aviv University, 69978 Tel Aviv, Israel

**Keywords:** Photosynthesis, Hydrogen production, Sustainability, Temperature-sensitive mutants, Photosystem II, *Chlamydomonas reinhardtii*

## Abstract

The need for energy and the associated burden are ever growing. It is crucial to develop new technologies for generating clean and efficient energy for society to avoid upcoming energetic and environmental crises. Sunlight is the most abundant source of energy on the planet. Consequently, it has captured our interest. Certain microalgae possess the ability to capture solar energy and transfer it to the energy carrier, H_2_. H_2_ is a valuable fuel, because its combustion produces only one by-product: water. However, the establishment of an efficient biophotolytic H_2_ production system is hindered by three main obstacles: (1) the hydrogen-evolving enzyme, [FeFe]-hydrogenase, is highly sensitive to oxygen; (2) energy conversion efficiencies are not economically viable; and (3) hydrogen-producing organisms are sensitive to stressful conditions in large-scale production systems. This study aimed to circumvent the oxygen sensitivity of this process with a cyclic hydrogen production system. This approach required a mutant that responded to high temperatures by reducing oxygen evolution. To that end, we randomly mutagenized the green microalgae, *Chlamydomonas reinhardtii*, to generate mutants that exhibited temperature-sensitive photoautotrophic growth. The selected mutants were further characterized by their ability to evolve oxygen and hydrogen at 25 and 37 °C. We identified four candidate mutants for this project. We characterized these mutants with PSII fluorescence, P700 absorbance, and immunoblotting analyses. Finally, we demonstrated that these mutants could function in a prototype hydrogen-producing bioreactor. These mutant microalgae represent a novel approach for sustained hydrogen production.

## Introduction

Photosynthesis is one of the most important life-sustaining reactions on our planet. It provides the energy required for the survival of all life forms, and it underlies the accumulation of fossil fuels, the main source of energy for sustaining modern human lifestyles (Barber 2004; Nelson and Yocum 2006). The evolution of photosynthesis is a prime example of a highly complex process that enabled the development of current life forms on earth. Photosynthesis was established on earth more than 3.5 billion years ago under anaerobic conditions (Falkowski and Isozaki 2008; Blankenship and Hartman 1998; Bendall et al. 2008). Oxygenic photosynthesis, which evolved shortly thereafter, used light energy and water to produce reducing equivalents. These equivalents could be used as chemical energy, and oxygen evolved as a by-product. About 1.5 billion years passed before metal compounds in the oceans and on the earth’s surface were oxidized, which allowed oxygen to accumulate in the atmosphere (Nelson 2011). During that period, oxygenic photosynthesis was limited by a shortage of oxidized electron acceptors, which are plentiful on the current earth surface. Arguably, protons served as a sink for the excess electrons, resulting in hydrogen molecules, most of which left earth, due to their light atomic weight, which allowed it to achieve escape velocity (11.2 km/s) at normal temperatures (Nelson 2011). Although this mechanism may not seem necessary in the present oxygen-rich atmosphere, it was discovered that some bacteria, microalgae, and cyanobacteria exhibited light-dependent hydrogen evolution (Gest and Kamen 1949; Lambert and Smith 1977; Amon et al. 1961). The enzyme responsible for this reaction uses electrons delivered by the photosynthetic electron transport chain (ETC), and this enzyme is highly sensitive to oxygen. Therefore, the reaction occurs strictly under anoxic conditions in the light. Within this framework, the notion of exploiting the photosynthetic apparatus to transform the most abundant energy source on our planet into the cleanest fuel is quite tempting (Kruse and Hankamer 2010).

Under strict anaerobic conditions, certain microalgae express the *HYDA* gene. This gene encodes a highly active, but oxygen-sensitive, [FeFe]-hydrogenase, which catalyzes the reversible reduction of protons to form H_2_ (Happe and Kaminski 2002). This hydrogenase is localized in the chloroplast stroma, coupled to the reducing site of the photosynthetic ETC (Winkler et al. 2009). The photosynthetic ETC is composed of several enzymes, most of which are embedded in the thylakoid membranes. The ETC is powered by light, which excites the reaction centers of Photosystem II (PSII) and Photosystem I (PSI). Light excitation of PSII results in the oxidation of water and the released electrons are transferred to PSI. When PSI is excited by light, these electrons are used to reduce ferredoxin (Nelson and Yocum 2006; Nelson and Ben-Shem 2004; Amunts and Nelson 2009; Nelson and Junge 2015). Ferredoxin may then supply reducing equivalents to [FeFe]-hydrogenase, which reduces protons to form H_2_ (Florin et al. 2001). PSI is indispensable for ferredoxin-dependent hydrogen production. Hydrogen production mainly takes place in the light (Redding et al. 1999). However, in the light, the hydrogenase is rapidly and irreversibly inactivated by PSII-induced O_2_ production (Ghirardi et al. 1997; Yacoby et al. 2011). PSII is not directly essential for hydrogen production, because electrons from other sources can be fed into the ETC by transferring them to the PQ-pool (Stuart and Gaffron 1972; Melis et al. 2000). However, the main non-photosynthetic electron source for reducing the PQ-pool is starch, which is produced by PSII-dependent photosynthesis (Hemschemeier et al. 2008).

Before we can even consider the option of exploiting this mechanism for mass hydrogen production, we must overcome three major challenges. The first challenge is to spatially separate oxygen and hydrogen production to prevent combustion to water and to promote the required anoxic conditions. The second challenge is to convert light energy efficiently, to ensure that the process is economically viable. The third challenge is the inevitable sensitivity of the target organism to external agents, such as chemicals, viruses, and stress conditions.

In an attempt to circumvent the oxygen sensitivity of photosynthetic hydrogen production, a system has been proposed that requires an organism with a temperature-sensitive PSII (Mazor et al. 2012). At low temperatures, this organism would perform regular oxygenic photosynthesis and accumulate starch; at high temperatures, PSII would be inactive, and anaerobic conditions would be sustained in the light. Then, hydrogen production could take place, provided that the photosynthetic electron flow is maintained by pumping electrons from starch through the plastoquinone. This kind of organism would be cycled between two growth chambers, with different temperatures; hence, it would alternate between aerobic biomass production and anaerobic hydrogen production.

In this study, we used random mutagenesis on the model organism, *C. reinhardtii*, to generate an organism with a temperature-sensitive PSII. Mutated *C. reinhardtii* cells with the required phenotype were selected, identified, and characterized. These mutants were able to produce hydrogen, and we constructed a prototype photosynthetic hydrogen-producing bioreactor.

## Materials and methods

### Random mutagenesis and screening

*C. reinhardtii* wild-type, strain 1A+, was grown in liquid TAP medium (Harris 2008) to mid-log phase. Then, 200–500 cells were plated on TAP agar plates. The plates were exposed to UV radiation for a time period sufficient to kill 80–90 % of cells. After UV exposure, plates were maintained in the light at 25 °C, until the surviving cells grew to visible colonies. Single colonies were resuspended in water and re-plated on three TAP agar plates. Of these, two plates lacked acetate (photoautotrophic) and were maintained in light, one at 25 °C and the other at 35 °C; the third plate was maintained in light at 35 °C. Strains that grew photoautotrophically at low temperature and only grew heterotrophically at high temperature were identified as temperature-sensitive photoautotrophic (TSP) mutants.

### Physiological measurements

*C. reinhardtii* wild-type strain and TSP mutants were grown photoheterotrophically in liquid TAP medium, on a shaker, with constant illumination (70 µmol m^−2^ s^−1^), and at either 25 or 37 °C. Cell density was monitored by measuring absorbance at 730 nm. The chlorophyll content was determined by acetone solubilization, as previously described (Arnon 1949).

The net oxygen exchange rate (OER) and oxygen respiration rate were measured with a Clark-type oxygen electrode (Oxygraph plus, Hansatech). An aliquot of cell culture (2 ml at a cell density of OD_730nm_ = 0.5–0.7) was transferred to the measuring chamber, and changes in oxygen concentration were measured over time, both at a light intensity of 300 µmol m^−2^ s^−1^ and in the dark. At the same time, the chlorophyll concentration in the cell culture was determined. The oxygen concentration changes were normalized to the chlorophyll concentration.

The maximum quantum efficiency of PSII (Fv/Fm) was calculated from in vivo chlorophyll fluorescence measurements, as previously described (Baker 2008). A JTS-10 spectrophotometer was used. An aliquot of cell culture (2 ml at a cell density of OD_730nm_ = 0.5–0.7) was incubated in the dark for 5 min and transferred into a measuring cuvette. Chlorophyll was excited with a low-intensity light (2 µmol m^−2^ s^−1^) at 520 nm, and fluorescence emission was measured at *λ* > 650 nm. This was performed before and immediately after illuminating the sample with a high-intensity light pulse of 2900 µmol m^−2^ s^−1^ at 520 nm for 250 ms.

The amount of P700 and the oxidized P700 half-life were calculated from in vivo oxidized P700 absorbance measurements with a JTS-10 spectrophotometer. An aliquot of cell culture (1 ml at a cell density of OD_730nm_ = 0.5–0.7) was incubated in the dark for 5 min and was transferred into a measuring cuvette. Absorbances of oxidized P700 were measured at 705 nm, first with no actinic light, then after illuminating the sample with a high-intensity light pulse of 3000 µmol m^−2^ s^−1^. The amount of P700 was expressed as the difference in absorbance measured before and immediately after the saturation pulse, normalized by the chlorophyll content. The development of absorbance after the saturation pulse was used to calculate the half-life.

### Immunoblotting

To prepare crude protein extracts, 1.5 ml of cell culture was centrifuged (2 min, 14,000×*g*, room temperature) and the pellet was resuspended in Laemmli protein buffer (65.8 mM Tris-HCl, pH 6.8, 2.1 % SDS, 26.3 % (w/v) glycerol, 0.01 % bromophenol blue). The final concentration corresponded to a chlorophyll content of 0.14 µg µl^−1^. Extracts were incubated at 65 °C for 30 min, then centrifuged (2 min, 14,000×*g*, room temperature) to pellet the cell debris. Supernatants were loaded onto a sodium dodecyl sulfate polyacrylamide gel electrophoresis (SDS-PAGE) to separate the proteins. The polyacrylamide gel comprised 5 and 16 % acrylamide sections for protein collection and separation, respectively. The separated proteins were transferred to a nitrocellulose membrane with a semi-dry transfer cell (Trans-Blot SD from Bio-Rad), according to the manufacturer’s instructions. The membrane was incubated for 1 h at room temperature in blocking buffer (20 mM Tris-HCl, pH 7.4, 0.5 M NaCl, 0.1 % Tween-20, 5 % (w/v) skim milk powder), then overnight at 4 °C in blocking buffer with a primary antibody, diluted 1:4000 (Liveanu et al. 1986). Primary antibodies were rabbit anti-D1 (reaction center protein) and rabbit anti-CP43 (antenna chlorophyll-binding protein). Next, the membrane was washed 3 times for 15 min in blocking buffer, then incubated for 1 h at room temperature in blocking buffer with the secondary antibody (peroxidase-conjugated goat-anti-rabbit IgG from Jackson ImmunoResearch), diluted 1:5000. This was followed by a second wash repeated 3 times for 15 min with blocking buffer, then once for 15 min with blocking buffer that lacked powdered milk. The peroxidase substrate (Western lightning Plus-ECL from Perkin Elmer) was added to the secondary antibody, according to the manufacturer’s instructions. The luminescent signal was detected on X-ray film. The primary antibodies were raised in rabbits injected with denatured D1 and CP43 proteins, which were electroeluted from PAGE gels (Liveanu et al. 1986).

### Hydrogen evolution assay

An aliquot of cell culture (15 ml at a cell density of OD_730nm_ = 0.5–0.7), with or without addition of a PSII inhibitor (10 mM DCMU), was transferred into a 25-ml glass bottle and sealed with a rubber cap. The bottle was flushed with argon for 1 min to deplete it of air, and then it was placed on a shaker in the light or in the dark. At different time points, 500 µl of the gas in the headspace was collected with a syringe and analyzed with a gas chromatograph (5890 Series II, Hewlett Packard). Simultaneously, 200 µl of cell culture was also collected with a syringe to determine the chlorophyll content. Over the period of hydrogen production, the average hydrogen production rates normalized to chlorophyll were calculated.

## Results

**Fig. 1:**
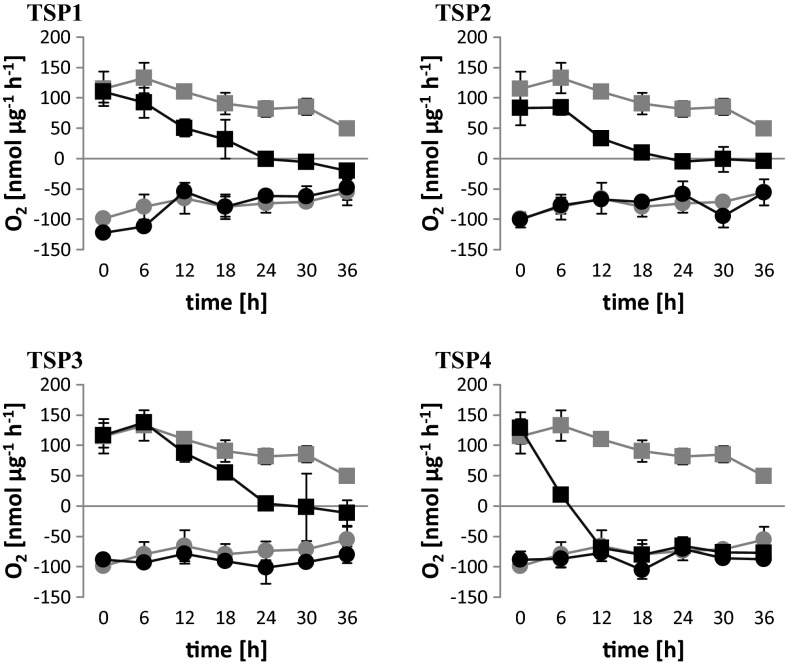
Oxygen exchange rates in *C. reinhardtii* wild-type and TSP mutants at 37 °C. All cultures were initially grown at 25 °C. In all charts, the *gray curves* correspond to the wild type; the *black curves* correspond to TSP1, TSP2, TSP3, and TSP4, as indicated. The light intensity was 60–70 μE m^−2^ s^−1^ during growth and during incubation at 37 °C. Measurements were performed in the dark (light intensity = 0; *circles*) and at a light intensity of 300 μE m^−2^ s^−1^ (*squares*). *Data* represent the mean ± SD of three independent experiments

*C. reinhardtii* cells were exposed to UV irradiation to induce random mutagenesis. Colonies (*n* = 12,000) grown from single cells were screened for temperature-sensitive photoautotrophic (TSP) mutants. We identified 157 TSP mutants, which were further characterized with physiological and biomolecular assays. Of these, four were selected as the most suitable: TSP1, TSP2, TSP3, and TSP4. The phenotypes of these mutants resembled the wild-type microalgae at 25 °C, but they differed from wild type at 37 °C. The oxygen exchange rates (OERs) of wild-type and mutant cultures were measured in the dark (0 μE m^−2^ s^−1^) and in the light (300 μE m^−^ s^−1^) during 37 °C incubations (Fig. 1). Over the course of 36 h, we measured oxygen respiration in the dark. In wild-type cultures, oxygen respiration decreased steadily from 99 to 55 nmol µg^−1^ h^−1^. All the mutant cultures showed similar declines in oxygen respiration in the dark. In the light, the net oxygen evolution in wild-type cultures also decreased over the course of 36 h, from 120 to 49 nmol µg^−1^ h^−1^. However, in the mutant cultures, oxygen evolution also declined, but at a significantly faster rate compared to wild-type cultures. The values fell below 0 nmol µg^−1^ h^−1^ after 24 h for TSP1, TSP2, and TSP3, and between 6 and 12 h for TSP4. Furthermore, TSP4 OERs were the same in the dark and in the light after 12-h incubations.

**Fig. 2:**
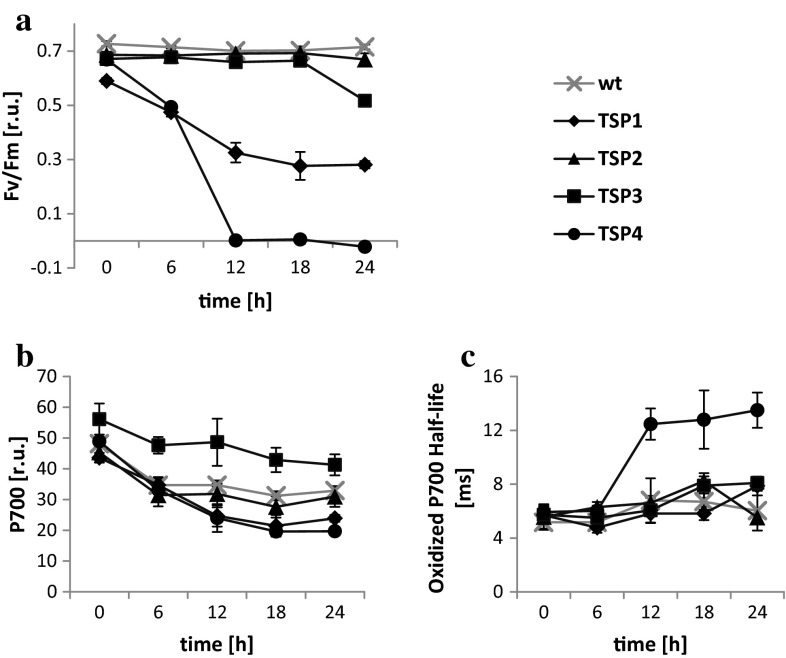
PSII and PSI reactions in *C. reinhardtii* wild-type and TSP mutants at 37 °C. **a** Maximum quantum efficiency of PSII, **b** amount of P700, and **c** oxidized P700 half-life; wild type is shown with *gray* crosses; mutant strains: TSP1 (*diamonds*), TSP2 (*triangles*), TSP3 (*squares*), and TSP4 (*circles*). All cells were incubated at 37 °C after growth at 25 °C. The light intensity was 60–70 μE m^−2^ s^−1^ during growth and during incubation at 37 °C. *Data* represent the mean ± SD of three independent experiments

To determine whether an absent PSII reaction center was responsible for the observed OER phenotype in mutants (Fig. 2a), we measured the maximum quantum efficiency of PSII with fluorescence spectroscopy over a 24-h period, during a 37 °C incubation. The PSII efficiency in TSP1 and TSP4 decreased by 50 and 100 %, respectively, after 12 h, and it remained stable thereafter. The PSII efficiency in TSP3 remained unaltered during the first 18 h and decreased by 25 % only after 24 h. The PSII efficiency in TSP2 remained unaltered over the entire period of incubation. Next, we measured P700 oxidation in response to a high-intensity light pulse, and we measured the subsequent reduction by determining light absorbance at 705 nm (Fig. 2b, c). At 12, 18, and 24 h of incubation at 37 °C, TSP1 and TSP4 had 30 % less P700 than wild-type algae. In contrast, TSP3 had 20 to 40 % greater amounts of P700 than wild-type microalgae over the whole period of incubation. The amount of P700 in TSP2 was similar to that in wild-type microalgae. The half-life of the fully oxidized P700 in the wild type was 5–8 ms, measured over the entire study period at 37 °C. Furthermore, TSP1, TSP2, and TSP3 showed half-lives similar to that of wild-type algae. However, TSP4 had a longer oxidized P700 half-life (12–14 ms) at 12, 18, and 24 h of incubation at 37 °C.

**Fig. 3:**
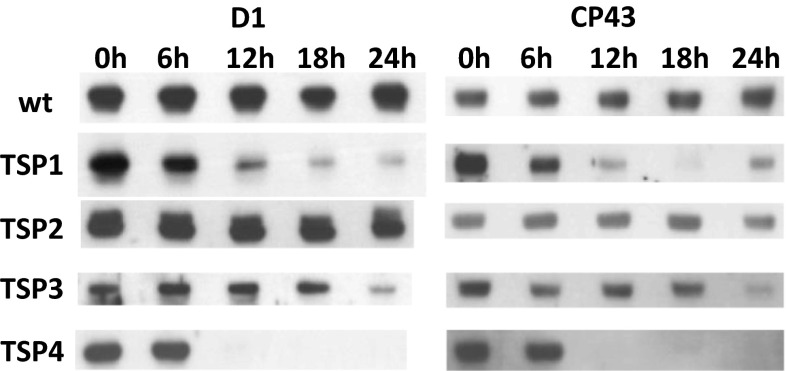
Immunoblots of PSII subunits, D1 and CP43, in *C. reinhardtii* cultures taken at different time points of adaptation to 37 °C. Wild-type (wt) and four mutant (TSP1, TSP2, TSP3, and TSP4) samples were assayed with antibodies against D1 (*left*) and CP43 (*right*). The amounts of sample loaded corresponded to 1.4 µg chlorophyll

We performed immunoblotting analyses to evaluate the levels of the PSII subunits, D1 and CP43, in each microalgae strain (Fig. 3). Wild-type and TSP2 cells maintained equal amounts of D1 and CP43 over the entire period of incubation. However, in TSP1 and TSP3, D1 and CP43 decreased after 12 and 24 h of incubation, respectively. In TSP4, both subunits were completely absent after 12 h of incubation.

**Fig. 4:**
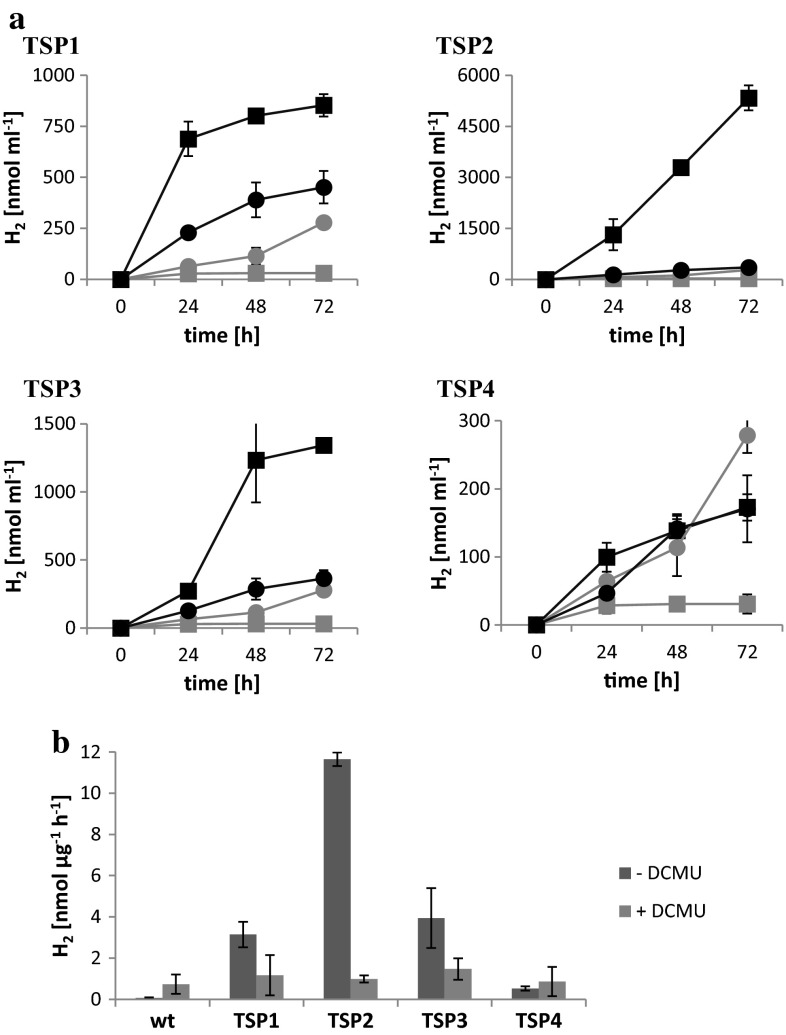
Hydrogen production in the light at 37 °C. **a** Hydrogen accumulation in sealed flasks per ml culture over 3 days. In all charts, the *gray* *curves* correspond to the wild type; the *black curves* correspond to TSP1, TSP2, TSP3, and TSP4, as indicated. The *squares* and *circles* represent cultures without and with DCMU, respectively. **b** Average hydrogen production rates during the most productive day. The values were normalized to chlorophyll content. The *dark* and *light bars* represent cultures without and with DCMU, respectively. All cultures were grown at 25 °C, incubated for 1 day at 37 °C, and then transferred to sealed bottles without and with 10 mM DCMU. The light intensity was 60–70 μE m^−2^ s^−1^ during growth and incubation at 37 °C. *Data* represent the mean ± SD of four independent experiments

To determine whether these mutants were capable of producing H_2_ and whether they could sustain H_2_ production over long time periods, the cultures were transferred to sealed, initially anaerobic flasks, and incubated in the light and in the dark. At different time points, the gas in the headspace of the flasks was measured with a gas chromatograph (GC). At 25 °C in the light, the wild-type and all four mutants accumulated oxygen in the flasks, and no significant hydrogen accumulation was detected. At 37 °C in the dark, no oxygen accumulated, and all cultures accumulated equal amounts of hydrogen over 24 h. The hydrogen production rate was 0.13 nmol µg^−1^ h^−1^. At 37 °C in the light, wild-type cultures accumulated oxygen, and no continuous hydrogen accumulation was detected. After 24 h, 30 nmol of hydrogen per ml culture was measured, and no significant increase was detected thereafter. Conversely, the mutants did not accumulate any oxygen, but accumulated continuously hydrogen (Fig. 4a). During 3 days, the mutants TSP1, TSP2, TSP3, and TSP4 accumulated 850, 5330, 1340, and 170 nmol hydrogen per ml of culture, respectively. If supplemented with the PSII inhibitor DCMU, all cultures including the wild type accumulated hydrogen continuously in the light. During 3 days, they produced 170–450 nmol hydrogen per ml culture. The average hydrogen production rate during the most productive day was calculated for all hydrogen-producing cultures (Fig. 4b). With no DCMU, these values were 3.1, 11.7, 3.9, and 0.5 nmol µg^−1^ h^−1^ for mutants TSP1, TSP2, TSP3, and TSP4, respectively. If supplemented with DCMU, the values of wild-type and mutant cultures were comparable and ranged between 0.5 and 1.5 nmol µg^−1^ h^−1^.

**Fig. 5:**
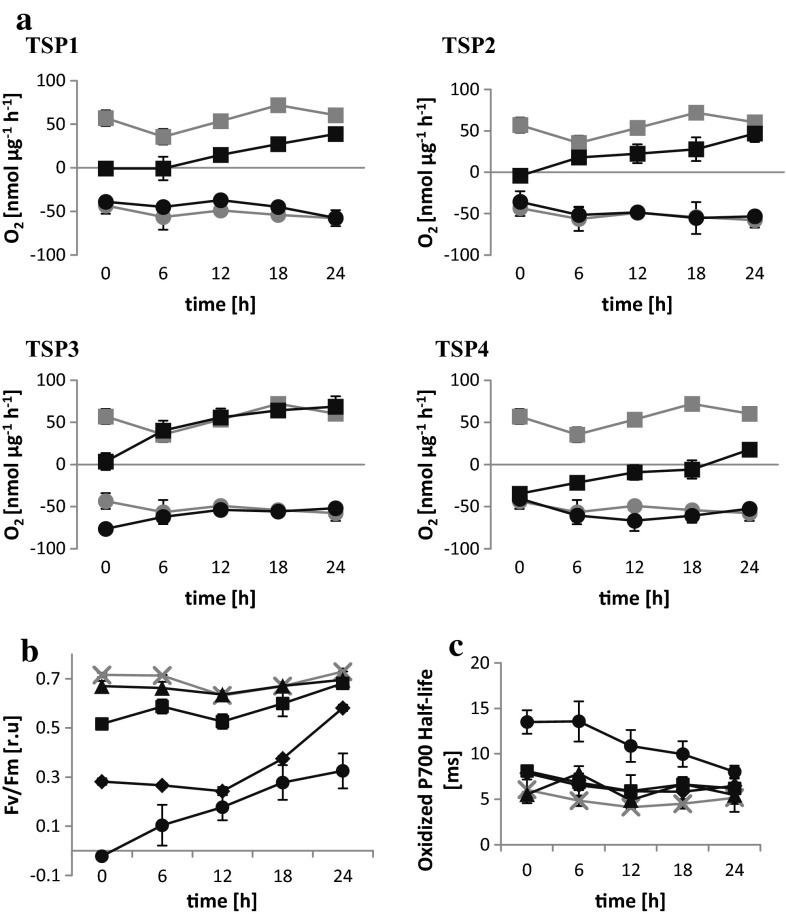
Physiological measurements of *C. reinhardtii* wild-type (*gray*) and TSP mutant (*black*) cultures during recovery at 25 °C, after 24-h incubations at 37 °C. **a** Oxygen exchange rate in the dark (*circles*) and in the light at 300 μE m^−2^ s^−1^ (*squares*); **b** maximum quantum efficiency of PSII; **c** oxidized P700 half-life. In **b** and **c**, TSP1, TSP2, TSP3, and TSP4 are represented by *diamonds*, *triangles*, *squares*, and *circles*, respectively. The light intensity was 60–70 μE m^−2^ s^−1^ during growth, at 37 °C, and during recovery at 25 °C. *Data* represent the mean ± SD of three independent experiments

Next, we tested mutant recovery to the wild-type phenotype. After 24-h incubations at 37 °C, mutant cultures were transferred back to 25 °C. Analogous measurements of OER, PSII fluorescence, and P700 absorbance were performed over a period of 24 h (Fig. 5). TSP1, TSP2, and TSP4 recovered steadily over the entire period; they reached 82, 100, and 64 % of wild-type OER values, respectively, after 24 h. TSP3 reached wild-type OER values after 6 h of incubation at 25 °C. Additionally, TSP1 and TSP4 reached 80 and 45 %, respectively, of wild-type PSII quantum efficiency. Furthermore, TSP4 fully recovered the oxidized P700 half-life of wild-type microalgae.

### Toward a photosynthetic cyclic hydrogen-producing bioreactor

**Fig. 6:**
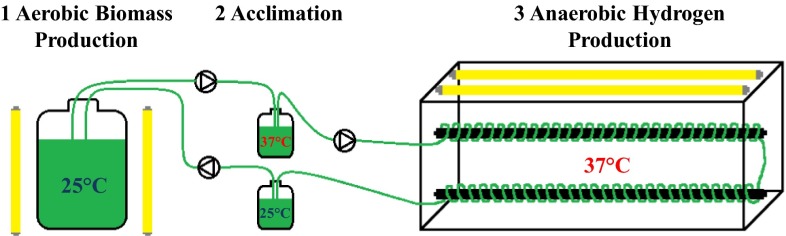
Cyclic photosynthetic hydrogen-producing bioreactor. The microalgal culture circulates between two illuminated main chambers. At 25 °C, the culture grows in an aerobic bottle (*1*); at 37 °C, the culture produces hydrogen in silicon tubing which passes through a sealed anaerobic collection chamber (*3*). While cycling from one chamber to the other, the culture is acclimated to the required temperature (*2*)

To fully assess the relevance of the presented mutants, their hydrogen production capability needs to be characterized in a cyclic bioreactor (Fig. 6). An important feature of our proposed hydrogen-producing bioreactor was the ability to exchange gases, particularly hydrogen, from the aqueous culture to the gas phase of the acclimated chamber. Therefore, we tested hydrogen production and diffusion through silicon tubes in a prototype bioreactor consisting only of the anaerobic hydrogen production chamber. The mutant TSP2 was adapted to 37 °C for 24 h and transferred to a sealed, anaerobic bottle. The adapted culture was pumped through the prototype bioreactor. The mutant could produce hydrogen in the silicon tubes, which diffused immediately into the anaerobic chamber, at the same rate achieved in a sealed bottle. This experiment set the stage for the design and implementation of a cyclic photosynthetic hydrogen-producing bioreactor, as we previously proposed (Mazor et al. 2012).

## Discussion

We identified four mutant microalgae strains that, compared to wild-type microalgae, exhibited a faster rate of decline in the ability to evolve oxygen, upon incubation at 37 °C. In contrast, these mutants maintained oxygen respiration at wild-type levels. Before the end of the 24-h incubation, all mutants, but no wild-type cultures, reached a point where oxygen uptake by respiration was higher than oxygen production. Thus, mutants exhibited net oxygen consumption in the light.

The net oxygen consumption condition is absolutely necessary for our proposed system to maintain anoxic conditions and enable hydrogen production. In fact, at 37 °C, all four mutants evolved hydrogen continuously in the light, over the period of 3 days. The average hydrogen production rates during the most productive day ranged between 0.5 and 11.7 nmol µg^−1^ h^−1^. With the addition of DCMU or in the dark, the hydrogen production rates were 0.5–1.5 or 0.13 nmol µg^−1^ h^−1^ in all mutant and wild-type cultures. Hence, the observed hydrogen production in the mutant cultures was mainly light dependent, and the hydrogenase received electrons through the photosynthetic ETC from residual PSII activity as well as from other sources. However, we observed different amounts of light-dependent hydrogen production among the various mutants, mainly due to a differential residual PSII contribution. This residual PSII activity raises the question of which reducing equivalent source was used for respiration to sustain anoxic conditions in the mutants. Acetate present in the media might have contributed to respiration. However, an acetate independent respiration would be desirable and starch accumulation remains to be elucidated.

Each of the selected TSP mutants exhibited unique properties. They showed different PSII inactivation time courses, different changes in the amounts of PSII subunits, and different levels of oxygen evolution recovery upon return to the permissive temperature. Mutant TSP1 exhibited a reduced ability to evolve oxygen in light at 37 °C over the entire incubation time. In addition, PSII fluorescence decreased to 60 % of wild-type levels, and D1 and CP43 also decreased to minimal amounts. The oxidized half-life of P700 was unaltered, which suggested that, despite the lower PSII activity, these cultures maintained electron transfer to PSI. However, the amount of P700 was 30 % lower than that in wild-type cultures. Nevertheless, PSII independent hydrogen production reached the highest value in this mutant, suggesting that PSI is not a limiting factor. Still, residual PSII activity was the main contributor for hydrogen production, at a lower rate compared to the TSP2 and TSP3 mutants.

In the TSP2 mutant, the OER decreased steadily, from 6 to 24 h of incubation at 37 °C, until it reached around 0 nmol µg^−1^ h^−1^, which was maintained thereafter. This scenario is optimal for our proposed system. Water oxidation continued to provide electrons for hydrogen production, but not at a rate that could compromise the maintenance of the required anoxic conditions. Fluorescence measurements and the detection of D1 and CP43 suggested that at least the core complex of PSII was not damaged at any point during the incubation. Furthermore, P700 levels are like in the wild type. These optimal conditions gave rise to the highest observed hydrogen production rate: 11.7 nmol µg^−1^ h^−1^.

In the TSP3 mutant, the OER decreased steadily, as observed in the TSP2 mutant, until it reached an OER of around 0 nmol µg^−1^ h^−1^ at 24 h; thereafter it decreased only minimally. PSII fluorescence remained at wild-type levels up to 18 h of incubation, and then declined by 25 % at 24 h. Immunoblots revealed reduced D1 and CP43 levels at 24 h of incubation. TSP3 had 20–40 % higher amounts of P700 than wild-type microalgae, over the period of incubation, and they exhibited the same half-life for oxidized P700. The higher amounts of P700 did not increase significantly the PSII independent hydrogen production rate, suggesting again that P700 is not a limiting factor. As well in this mutant, PSII was the main contributor for hydrogen production. The productivity was somewhat higher than in the TSP1 mutant and quite lower than in the TSP2 mutant. This suggests a further decline of PSII after the adaptation time of 24 h.

In the TSP4 mutant, the OER decreased to almost 0 nmol µg^−1^ h^−1^ after only 6 h of incubation; after 12 h, oxygen production was completely absent. PSII fluorescence was also completely absent, starting at 12 h and continuing throughout the study period. D1 and CP43 levels were practically undetectable on immunoblots. This most severe phenotype had also an effect on P700 reduction. After 12 h of incubation, the half-life of oxidized P700 was 12–14 ms, or about twice the half-life observed in wild-type microalgae. Furthermore, the amount of P700 decreased by 30 % after 12 h of incubation. These features resulted in a relatively low hydrogen production rate (0.5 nmol µg^−1^ h^−1^). Nevertheless, this rate was higher than the rate achieved in the dark. Hence, although no electrons could be delivered from water oxidation, PSI delivered electrons for hydrogen production.

A crucial element in our proposed cyclic hydrogen production system is the ability of mutants to return to the wild-type photosynthetic phenotype after a cycle of hydrogen production. This feature was achieved in all four mutants; after 24 h at 37 °C, all mutants recovered the wild-type phenotype upon incubation at 25 °C.

Several previous studies were conducted to establish an economically viable method for large-scale photosynthetic hydrogen production systems. Many focused on generating an O_2_-insensitive hydrogenase (Bandyopadhyay et al. 2010; Burgess et al. 2011). That system would be beneficial, because it includes PSII to supply a maximal amount of electrons for hydrogen production. However, no significant decrease in O_2_ sensitivity has been achieved; furthermore, that system would not include the necessary spatial separation of oxygen and hydrogen to prevent combustion.

The most common protocol for achieving sustained H_2_ production in the light was sulfur starvation (Melis et al. 2000; Melis and Happe 2001). In that protocol, cultures are grown normally under aerobic conditions, and then they are centrifuged and resuspended in sulfur-depleted media. Upon sulfur deprivation, cells are unable to synthesize proteins that contain sulfur, like D1. Because the D1 protein of PSII has a short half-life, it must be continuously replaced by newly synthesized D1 to ensure PSII activity. Therefore, this protocol causes a reduction in PSII activity over the first 20–30 h, but oxygen respiration is maintained (Kosourov et al. 2002). As a result, anoxic conditions are reached in a closed environment, and hydrogen production takes place over several days, with maximal hydrogen production rates of 4–6 nmol µg^−1^ h^−1^ under standard conditions and up to 9.4 nmol µg^−1^ h^−1^ under optimized growth conditions (Kosourov et al. 2003). Eventually, the cells die from sulfur starvation. In that system, the reduction in PSII activity causes a reduction in energy conversion efficiency; however, it enables sustained hydrogen production over a long period of time.

It has been calculated that biophotolytic energy conversion efficiencies must be around 5 % for economic viability (Kruse et al. 2005); currently, efficiencies of around 1 % have been achieved with sulfur deprivation (Kruse et al. 2005). Many different approaches to the sulfur starvation system have been investigated in endeavors to increase hydrogen production efficiencies (Burgess et al. 2011). Those studies raised the hope that 5 % energy conversion efficiencies might be achievable in that system. However, the system may not be realizable at large scales, due to the impracticality of transferring cells into sulfur-depleted media. Furthermore, the cell death caused by sulfur starvation limits this system to a batch process. Therefore, direct regulation of PSII in a controlled, simple manner would be more practical in an analogous system. Rochaix and colleagues attempted the latter approach with an inducible promoter. That protocol proved to be an effective method for controlling hydrogen production without inducing fatal stress to the cells; thus, cyclic hydrogen production is achievable in that system (Surzycki et al. 2007). However, the promotor was induced by copper; therefore, cell transfer between copper-containing and copper-free media remains an obstacle for upscaling the protocol.

The present study described a system that employed TSP mutants and induction of anoxic conditions by increasing the incubation temperature. These conditions triggered hydrogen production at the same rates achieved by the sulfur starvation protocol. However in our system, cells were not exposed to deadly stress, and they could be recycled by simply reducing the temperature. Thus, these TSP mutants provide a convenient platform for increasing hydrogen production by physical means to achieve 5 % energy conversion efficiencies in a platform that is amenable to upscaling.

The upscale system would require two compartments: one for accumulating biomass and the other for producing hydrogen. The first compartment could be an open pond, which is currently used industrially to grow *Dunaliella* for producing B-carotene (Ben-Amotz et al. 1989; Oren 2014). The second compartment should be a tubular reactor composed of transparent, H2-permeable tubing. This reactor must be enclosed in a translucent, airtight chamber. The second compartment was successfully tested, on a small scale, in the present study. In the absence of air convection, the temperature could be increased in the second compartment without an external energy source; this feature could facilitate the development of the mutant phenotype and allow efficient hydrogen production. Hydrogen then could diffuse through the tubing and accumulate in the airtight compartment for collection.

## Conclusion

In this work, we created TSP mutants of *C. reinhardtii* that could alternate between oxygenic photosynthesis and anoxic hydrogen production, in response to changes in temperature. This approach represented a solution to the challenge posed by the oxygen sensitivity of the [FeFe]-hydrogenase. Our protocol was an analogous, however viable, alternative to the sulfur starvation protocol for sustained hydrogen production. Consequently, these mutants are an adequate system for upscaling sustained hydrogen production. Studies that aim to increase energy conversion efficiencies in sulfur-starved cells might be tested with these TSP mutants. Therefore, they represent an alternative platform for overcoming the obstacle of non-economically viable energy conversion efficiencies. Finally, the proven quality of *C. reinhardtii* as a model organism for industrial endeavors provides confidence that the developed technology can be readily transferred to a mass hydrogen production plant.
